# Efficacy of ultraviolet germicidal irradiation (UVGI) devices to decrease the incidence of respiratory infections in nursing homes: study protocol for a cluster randomized crossover trial (RESPROTECT)

**DOI:** 10.1186/s13063-026-09797-y

**Published:** 2026-05-20

**Authors:** Cyril Cornille, Eric Luneau, Delphine Gabillard, Mickael Mathieu, Sophie Lengagne, Estelle Perrin, Myriam Ezzouak, Frédérique Gondol, Marie Peuriere, Bertrand Maubert, Pierre Saint-Sardos, Sabine Peghaire, François-Xavier Blanc, Didier-Koumavi Ekouevi, Xavier Anglaret, Emilie Gadea

**Affiliations:** 1 Department of General Medicine, Emile Roux Hospital, Le Puy en Velay, France; 2 Clinical Research Unit, Emile Roux Hospital, Le Puy en Velay, France; 3 Bordeaux Population Health, University of Bordeaux, Inserm UMR1219, IRD EMR 271, Bordeaux, France; 4 Laboratory of Microbiology, Emile Roux Hospital, Le Puy en Velay, France; 5 Department of Hygiene, Emile Roux Hospital, Le Puy en Velay, France; 6 Service de Pneumologie, Institut du Thorax, Nantes Université, CHU de Nantes, Nantes, France

**Keywords:** Ultraviolet germicidal irradiation, UVGI, Respiratory infections, Nursing homes, Elderly care, Cluster randomized trial, Crossover design, Infection prevention

## Abstract

**Background:**

Ultraviolet germicidal irradiation (UVGI) has long been recognized for its ability to inactivate airborne pathogens under experimental conditions. However, despite strong in vitro evidence, randomized clinical evidence of its real-world effectiveness remains lacking. Nursing homes—where respiratory infections are a major cause of morbidity and mortality—represent a highly relevant setting to evaluate the preventive potential of UVGI. The RESPROTECT trial aims to assess whether continuous UVGI use in communal areas of nursing homes reduces the incidence of severe acute respiratory infections (ARIs) among residents.

**Methods:**

RESPROTECT is a multicenter, open cohort, quadruple-blind, cluster-randomized, crossover trial. A total of 848 UVGI devices were installed in communal living areas of 12 nursing homes in the Haute-Loire region, France. All devices are switched on, but some contain internal filters that deactivate UV light while remaining indistinguishable from the outside. Centers were randomized 1:1 into two groups: unfiltered UVGI devices active during period 1 (October 2024–April 2025), then filtered (inactive) during period 2 (October 2025–April 2026), or the reverse sequence. The primary outcome is the incidence of severe ARIs. Secondary outcomes include the incidence of ARIs of any grade, all-cause hospitalization or death, adverse events of interest (keratitis and erythema), antibiotic consumption, airborne and surface pathogen loads, and cost-effectiveness. All analyses will follow the intention-to-treat principle and use mixed-effects Poisson regression models with fixed effects for period and random effects for clusters, cluster-periods and individuals.

**Discussion:**

RESPROTECT is one of the first large-scale blinded randomized trials to evaluate the clinical effectiveness of UVGI in preventing respiratory infections among elderly residents in nursing homes. It will complement recent findings from the PETRA trial (*JAMA Intern Med* 2025) and is designed to provide robust data on both clinical outcomes and environmental contamination. If UVGI proves effective, it could represent a practical, low-maintenance, and scalable infection-prevention strategy for high-risk institutional settings.

**Trial registration:**

French RCB identifier: RECHMIE-22-0003 - 2024-A00199-38. ClinicalTrials.gov Identifier: NCT06569160 (Registered on August 21, 2024). https://clinicaltrials.gov/study/NCT06569160.

## Administrative information

**Table:** 

Title {1}	Efficacy of Ultraviolet Germicidal Irradiation (UVGI) devices to decrease the incidence of respiratory infections in nursing homes: a cluster randomized crossover trial (RESPROTECT)
Trial registration {2a and 2b}	The trial is registered at ClinicalTrials.gov: NCT06569160
Protocol version {3}	Version 3.0, dated Septembre 30,2025
Funding {4}	The trial is funded by the French National Agency on AIDS and Emerging Infectious Diseases reseach (ANRS-MIE)
Author details {5a}	Cyril CORNILLE^1,2^, Eric LUNEAU^2^, Delphine GABILLARD^3^, Mikael MATHIEU^2^, Sophie LENGAGNE^2^, Estelle PERRIN^2^, Myriam EZZOUAK^2^, Frédérique GONDOL^2^, Marie PEURIERE^2^, Bertrand MAUBERT^4^, Pierre SAINT-SARDOS^4^, Sabine PEGHAIRE^5^, François-Xavier BLANC^6^, Didier-Koumavi EKOUEVI^3^, Xavier ANGLARET^2,3^, Emilie GADEA^2^ 1 Department of General Medicine, Emile Roux Hospital, Le Puy en Velay, France 2 Clinical Research Unit, Emile Roux Hospital, Le Puy en Velay, France 3 Bordeaux Population Health, University of Bordeaux, Inserm UMR1219, IRD EMR 271, Bordeaux, France 4 Laboratory of microbiology, Emile Roux Hospital, Le Puy en Velay, France 5 Department of Hygiene, Emile Roux Hospital, Le Puy en Velay, France 6 Service de Pneumologie, Institut du thorax, Nantes Université, CHU de Nantes, Nantes, France
Name and contact information for the trial sponsor {5b}	Centre Hospitalier Emile Roux 12 Bd. du Dr. Chantemesse, 43000 Le Puy-en-Velay, France Contact: Emilie Gadea, emilie.gadea@ch-lepuy.fr
Role of sponsor {5c}	The sponsor approved the study protocol and oversees trial monitoring. It ensures compliance with regulations and good clinical practice, as well as participant safety. It has no role in the interpretation of results

## Introduction

### Background and rationale {6a}

Acute respiratory infections (ARIs) are among the most frequent causes of morbidity and mortality in older adults, particularly in nursing homes where communal living in confined environments favors the spread of airborne pathogens [1]. In French nursing homes, the incidence of lower respiratory tract infections is estimated at 1 episode per 1000 resident-days—a rate 30 times higher than in the general population and 10 times higher than among community-dwelling individuals aged 75 years and older [2].

The vulnerability of nursing home residents arises from multiple factors, including advanced age, comorbidities, and immunosenescence. Age-related immune decline is associated with impaired innate and adaptive immune responses, leading to reduced pathogen clearance and weaker vaccine responses [3]. The high prevalence of comorbid conditions such as chronic obstructive pulmonary disease, cardiovascular disease, diabetes, and chronic kidney disease further increases the risk of both contracting ARIs and experiencing poor outcomes [4]. As a result, mild or moderate ARIs lead to discomfort, increased healthcare utilisation, and frequent prescriptions of antibiotics or other drugs [5]. More severe ARIs, such as pneumonia, are a leading cause of hospitalization and death among nursing home residents [6, 7].

Preventive strategies for respiratory infections in nursing homes currently rely on a combination of non-pharmacological interventions. These include mask use during outbreaks or by symptomatic individuals [8], strict hand hygiene practices [9], isolation of suspected or confirmed cases [10], restriction of visits during epidemic periods [11], and enhancement of natural or mechanical ventilation [12]. While these measures have shown benefits, their effectiveness may be limited by practical constraints, variable adherence, and infrastructural limitations.

Ultraviolet germicidal irradiation (UVGI), particularly upper-room UVGI, has emerged as a potentially valuable complementary approach. UVGI systems use short-wave ultraviolet light (UV-C, typically 254 nm) to inactivate airborne microorganisms. Laboratory studies have consistently demonstrated that UVGI rapidly inactivates a wide spectrum of viruses and bacteria, including influenza and coronaviruses, after very short exposure times [13, 14]. More recently, Fischer et al. showed that UV-C light completely blocked aerosol transmission of highly contagious SARS-CoV-2 variants in animal models [15]. Historical studies, some dating back to the mid-twentieth century, suggested reductions in respiratory illness incidence in institutional environments equipped with UVGI, but most of these studies were non-randomized and conducted with older devices [16, 17]. As a result, UVGI has not been widely recommended in countries such as France.

The COVID-19 pandemic highlighted the extreme vulnerability of nursing home residents. In a Spanish cohort study, the cumulative incidence of COVID-19 among residents reached 35%, with nearly 9% mortality during the early pandemic phase [18]. Similar findings were reported in French nursing homes, where factors such as advanced age, comorbidities, building design, and staffing ratios were associated with higher infection and mortality rates [19]. These observations have renewed interest in environmental interventions to reduce airborne transmission in closed institutional settings.

Taken together, the evidence underscores a major gap: while UVGI is biologically plausible and supported by strong laboratory data, there is a lack of contemporary randomized controlled trials in real-world nursing home environments. At a time when airborne epidemics are becoming more frequent and the population of nursing home residents is increasing, robust evidence is needed to determine whether UVGI should be recommended on a large scale.

### Objectives {7}

The primary objective is to compare the incidence of severe ARIs between periods when nursing homes are equipped with active UVGI systems and periods when the UVGI systems are inactive.

The secondary objectives are to compare, between periods with active and inactive UVGI, the following outcomes:

- Incidence of ARIs of any severity.
- Incidence of all-cause hospitalization or death.
- Incidence of adverse events of interest (AEIs)(erythema in skin areas exposed to light and keratitis), overall and by severity grade.
- Antibiotic consumption.
- Presence of respiratory pathogens in air samples and on fomites.
- Potential medico-economic benefits associated with the installation of UVGI systems.

### Trial design {8}

This is a multicenter, cluster-randomized, crossover trial conducted in an open cohort of nursing home residents. The study is designed as a quadruple-blind, placebo-controlled trial.

## Methods: participants, interventions, and outcomes

### Study setting {9}

The study is conducted in 12 nursing homes (Établissements d’Hébergement pour Personnes Âgées Dépendantes, EHPAD) located in the Haute-Loire department, France (list of facilities available in the Acknowledgments section).

### Eligibility criteria {10}

#### Inclusion criteria

##### Inclusion of centers:

To be eligible, participating nursing homes must:

- Have a system in place for monitoring cases of upper and lower ARIs among residents.
- Provide written agreement to participate in the study and comply fully with the study protocol.
- Accept the installation of UVGI devices.
- Commit to informing all residents and, where applicable, their legal representatives.

##### Inclusion of individuals:

All residents present for at least one day in a participating nursing home during either of the two predefined study periods are eligible. The study periods are:

- Period 1: October 1, 2024 – April 30, 2025
- Period 2: October 1, 2025 – April 30, 2026

#### Exclusion criteria

##### Exclusion of centers:

Nursing homes will be excluded if any of the following criteria are met:

- The facility is already equipped with another air treatment system in shared living areas;
- The facility declines to install UVGI devices in all rooms deemed necessary by the coordinating investigator;
- Major structural changes (e.g., renovation, extension, closure, or functional requalification) are planned during the study period that could affect the study population and/or interfere with the UVGI systems.

##### Exclusion of individuals

Residents of participating centers are excluded if, at any time between the start of the study and the analysis of results, they or their legal representatives express the wish that their personal data not be used for the study.

### Who will take informed consent? {26a}

This is an opt-out study, conducted in accordance with Article L1122-1–4 of French Law No. 2012-300 of March 5, 2012. All residents of participating nursing homes receive an information notice written in accessible language, detailing the study’s objectives, methodology, expected benefits, and potential constraints. The notice is also distributed to their relatives and, where applicable, their legal representatives.

The notice explicitly informs residents and their representatives of their right to object, at any time, to the use of their personal data for research purposes. Nursing staff and members of the study coordination team are available to answer any questions residents or their representatives may have.

No data is collected for any resident who, or whose representative, expresses opposition to participation. If an opt-out request is made after data collection has already begun, all previously collected personal data is permanently deleted.

### Additional consent provisions for collection and use of participant data and biological specimens {26b}

No biological specimens are collected from participants as part of this trial.

## Interventions

### Explanation for the choice of comparators {6b}

Each participating nursing home serves as its own control through the sequential use of active and inactive UVGI systems. The use of inactive UVGI systems as a comparator allows control for any potential effect related to the visible presence of the devices.

### Intervention description {11a}

UVGI devices are installed in all participating centers prior to the start of the study by a team of independent contractors, following standardized installation procedures. The installation team performs a commissioning visit before the start of the first study period and a maintenance visit during the washout period preceding the second period.

The UVGI devices are switched on at the beginning of each study period and switched off the day following its end. During each period, the devices remain continuously operational.

UV inactivation filters are installed in nursing homes allocated to arm B during the commissioning visit and removed during the maintenance visit; conversely, they are installed in nursing homes allocated to arm A during the maintenance visit.

To maintain blinding, residents, nursing homes staff, investigators, and members of the clinical trial unit responsible for data collection and processing remain unaware of the presence or absence of the filters.

### Criteria for discontinuing or modifying allocated interventions {11b}

The intervention may be temporarily or permanently discontinued in a given nursing home under specific circumstances, including:

- Safety concerns related to the devices (e.g., malfunction, overheating);
- Major unforeseen structural works (e.g., emergency ceiling repairs);
- Formal opposition expressed by the facility administration or by a substantial number of residents or their legal representatives.

Any interruption or modification of the intervention will be documented and immediately reported to the coordinating center. Decisions regarding discontinuation will be made by the study sponsor.

### Strategies to improve adherence to interventions {11c}

Periodic technical inspections are carried out by the technical team to verify device functionality and ensure compliance with planned activation periods. Additionally, staff at participating nursing homes receive briefings on study procedures and are instructed not to tamper with the devices.

### Relevant concomitant care permitted or prohibited during the trial {11d}

At the individual level, participating residents may be enrolled in other interventional or observational studies, provided these studies do not influence the incidence, diagnosis, treatment, or management of respiratory infections. Outside the research context, all routine medical care remains permitted. Once a resident’s participation in RESPROTECT ends—whether due to scheduled termination or early discharge—participation in other research projects may resume without restriction.

At the nursing home level, no concomitant intervention is specifically prohibited, except for the introduction of environmental air treatment systems (e.g., HEPA filtration, ionization) that could interfere with the evaluation of the UVGI devices.

### Provisions for post-trial care {30}

At the individual level, no specific post-trial care is planned.

At the nursing home level, nursing homes administrations will decide whether to continue using UVGI devices after trial completion, depending on the trial results and recommendations from health authorities.

### Outcomes {12}

#### Primary outcome

The primary outcome is the incidence of severe upper and lower ARIs occurring in residents of participating nursing homes during the study periods.

Severe infections are defined as those leading to oxygen therapy or followed within 30 days by hospitalization or death. Infections considered include sinusitis, otitis media, nasopharyngitis, pharyngitis, tonsillitis, tracheitis, bronchitis, pulmonary abscess, pneumonia, and acute exacerbations of chronic obstructive pulmonary disease (COPD).

Documented symptomatic infection with SARS-CoV-2, influenza virus, or respiratory syncytial virus is classified as a respiratory infection by default, even in cases presenting primarily with extra-respiratory symptoms.

### Secondary outcomes

Secondary outcomes include:

- Incidence of symptomatic ARIs of any severity.
- Incidence of non-infectious AEIs, namely keratitis and erythematous skin eruptions in light-exposed areas, overall and stratified by severity according to the *Common Terminology Criteria for Adverse Events* (CTCAE).
- Incidence of all-cause hospitalization or death, analyzed separately and combined.
- Total antibiotic consumption, overall and by antibiotic class.
- Viral load in air samples collected in communal living areas.
- Viral, bacterial and fungal load on surface (fomite) samples collected in communal living areas.
- Incremental cost-effectiveness ratio of UVGI implementation.

### Participant timeline {13}

The participant follow-up timeline is summarized in Table 1. This schedule outlines key study activities across all phases—pre-trial preparations, the two intervention periods, and the inter-period washout. All participating nursing homes and their residents are followed throughout the entire study to ensure consistent data collection and adherence to the study protocol.

**Table 1:** Participant timeline: schedule of enrollment, interventions, and assessments

**Activity**	**Pre-trial (Sept 2024)**	**Period 1 (Oct 2024–Apr 2025)**	**Wash-out (May 2025–Sept 2025)**	**Period 2 (Oct 2025–Apr 2026)**
Individual information of residents	X	X	X	X
Collection of opt-out requests	X	X	X	X
UVGI system implemented	X			
UVGI systems filtered according to randomization	0/X		X/0	
UVGI systems turned on		X		X
UVGI systems turned off	X		X	
Collection of baseline data of residents	X	X	X	X
Monitoring of ARIs^a^ and AEIs^b^		X		X
Monitoring of hospitalizations, and deaths		X	X	X
Pathogen sampling on air and surface fomites		X		X

^a^*ARIs*: upper (sinusitis, otitis media, nasopharyngitis, tonsillitis, tracheitis) and lower (bronchitis, pneumonia, and COPD exacerbations) respiratory tract infections. Documented symptomatic infection with SARS-CoV-2, influenza, or RSV is considered a respiratory infection by default, even if presenting with predominant extra-respiratory symptoms

^b^*AEIs*: adverse events of interest (keratitis and erythema of the skin in light-exposed areas)

### Sample size {14}

Given the open-cohort design, participants who die or permanently leave the nursing home are replaced by new residents, ensuring a stable number of participants per cluster over time. It is anticipated that approximately 80% of participants will be present during both study periods.

The within-period intra-cluster correlation coefficient (WP-ICC, *ρ*) is estimated at 0.1, indicating that approximately 10% of the variance in ARI incidence is attributable to nursing home-level effects. The between-period intra-cluster correlation (*η*), reflecting inter-period correlation within clusters, is considered negligible because the two periods correspond to same winter seasons separated by one year. A cluster autocorrelation of 0.5 was assumed (*η* = 0.05), consistent with recommendations for crossover trials with few clusters [20]. The individual autocorrelation was set at 0.2.

Assuming an ARI incidence rate of 17 per 100 person-years when UVGI devices are inactive, 12 clusters will provide 80% power to detect a 50% reduction in ARI incidence, using a two-sided *α* of 0.05 [21, 22].

### Recruitment {15}

Participating nursing homes were selected based on their capacity to ensure that the overall number of participants meets the sample size required for adequate statistical power. All residents present in participating nursing homes during either study period are eligible, provided they have not expressed opposition to participation.

## Assignment of interventions: allocation

### Sequence generation {16a}

Centers were randomly allocated to one of two arms (A or B) in a 1:1 ratio using a centralized computerized randomization system. Randomization was stratified by geographical area to ensure balanced distribution of centers with respect to environmental factors.

### Concealment mechanism {16b}

Randomization was conducted by an independent individual not involved in the conduct of the study. This person is affiliated with the technical team responsible for UVGI equipment installation and is bound by confidentiality obligations. Allocation information was concealed until the time of implementation.

### Implementation {16c}

The allocation of each center determined whether internal UV inactivation filters are installed during the commissioning or maintenance visits. The installation team implements the allocation as per the randomization schedule, without disclosing any information to investigators, staff, or residents.

## Assignment of interventions: blinding

### Who will be blinded {17a}

All UVGI devices operate continuously during intervention periods, and the presence or absence of internal filters is not externally visible. Consequently, members of the clinical research unit, investigators, participants, and nursing homes staff remain blinded to allocation.

Data on ARIs, AEIs, hospitalizations, and deaths—including their causes—are recorded and monitored by blinded personnel. Members of the Scientific Advisory Board (SAB) receive only aggregated progress reports without arm-specific details until final analysis. Members of the Data and Safety Monitoring Board (DSMB) receive the randomization list directly from the independent individual responsible for randomization. The DSMB is the only body authorized to access arm-specific reports before the final analysis. All DSMB members sign a Non-Disclosure Agreement to preserve the integrity of blinding.

### Procedure for unblinding if needed {17b}

Although highly unlikely given the well-documented safety profile of UVGI systems [23–25], unblinding may be authorized by the study sponsor in the event of a suspected safety concern. Such a request must be supported by a justified suspicion of a causal link between a morbid event and UV irradiation.

## Data collection and management

### Plans for assessment and collection of outcomes {18a}

The trial involves the collection of clinical outcomes (ARIs, AEIs, all-cause hospitalizations, and all-cause deaths) and microbiological endpoints (microbial loads in air and surface samples from nursing home communal areas).

#### Clinical outcomes

The occurrence of ARIs, AEIs, all-cause hospitalizations, and all-cause deaths is reported weekly to the trial’s clinical research assistants (CRAs) by a designated contact person at each center. CRAs verify individual reports against predefined diagnostic criteria and classify each event as validated (criteria met), rejected (criteria not met), or deferred (uncertain classification pending review).

A Clinical Event Review Committee (CERC), composed of the investigating physicians, meets weekly to adjudicate deferred events and systematically review all hospitalization and death records.

CRAs and the CERC have secure online access to the electronic care records of participating nursing homes and to hospital records for hospitalized residents.

To ensure completeness of event collection, the nursing home software is queried every three months to generate comprehensive lists of all discharges (death, hospitalization, return home, or transfer to another facility), as well as oxygen prescriptions. These data are cross-checked against the study database, and any missing or inconsistent events are investigated and adjudicated by the CERC, in accordance with best practices for independent endpoint review committees [26].

#### Microbiological outcomes

Environmental monitoring is performed to quantify pathogen contamination in the air and on surfaces within nursing home communal areas. Air and surface samples are collected at baseline and every seven weeks during both study periods.

Air samples are obtained using volumetric air samplers operating at standardized flow rates. Surface samples are collected using tryptic soy agar (TSA) contact plates and sterile polyester-headed swabs (eSWAB) applied to predefined fomites. Two self-adhesive plastic fomites are installed in each center—one in a dining room and one in a communal activity room.

Air samples are analyzed by quantitative PCR (qPCR) assays targeting SARS-CoV-2, influenza A and B viruses, and respiratory syncytial virus, using the *Triplex Thermo Fisher TaqPath COVID-19, FluA/B, RSV Combo Kit* (Ref. A49867). Surface samples are processed for bacterial and fungal detection by culture on TSA plates, followed by species identification using matrix-assisted laser desorption/ionization time-of-flight mass spectrometry (MALDI-TOF MS, Bruker Daltonics). Viral detection from fomites is performed by qPCR.

### Plans to promote participant retention and complete follow-up {18b}

Participant retention and complete follow-up are inherently supported by the continuous residency of participants within each nursing home.

Additionally, linkage with nursing homes software minimizes the risk of missing data, as transfers, hospitalizations, and deaths are automatically captured and reconciled with the study database.

### Data management {19}

Study data are entered into a secure electronic case report form (eCRF) hosted on a certified platform compliant with French regulations, the European General Data Protection Regulation (GDPR), and international Good Clinical Practice (GCP) standards.

Data entry is performed by trained CRAs. The eCRF system includes automated validation rules such as range checks, mandatory fields, and internal consistency controls.

Data quality is ensured through internal and external monitoring procedures at key trial stages, in line with ICH-GCP guidelines [27].

- *Internal monitoring* involves regular database queries to identify missing, inconsistent, or outlier data.
- *External monitoring* includes on-site source data verification and compliance checks with regulatory and ethical standards.

### Confidentiality {27}

All data are pseudonymized using coded identifiers. No directly identifiable information is stored in the study database.

Investigators and clinical research staff are trained in GCP and have signed confidentiality agreements covering all personal data from source documents, database records, and analytical outputs.

Access to the database is restricted to authorized users via secure, traceable logins, and data transfers are encrypted.

### Plans for collection, laboratory evaluation, and storage of biological specimens for genetic or molecular analysis in this trial/future use {33}

No biological specimens from participants are collected, analyzed, or stored for future genetic or molecular analyses as part of this trial.

## Statistical methods

### Statistical methods for primary and secondary outcomes {20a}

#### General approach

All analyses will follow the intention-to-treat (ITT) principle, including all centres and residents present during the study, regardless of duration of follow-up or early discharge, excluding only individuals who opted out. A two-tailed *p*-value < 0.05 will be considered statistically significant.

Each participant has their own baseline date for each period:

- For period 1: October 1, 2024, if the participant was already present on October 1 st, 2024; The date of entry to the ENH if the participant arrived at the ENH between October 2, 2024, and April 30, 2025.
- For period 2: October 1, 2025, if the participant was already present on October 1 st, 2025; The date of entry to the ENH if the participant arrived at the ENH between October 2, 2025, and April 30, 2026.

Each participant has their own end-of-follow-up date for each period:

- For period 1, the end of follow-up of a participant is April 30, 2025, or the date of premature end of follow-up in period 1.
- For period 2, the end of follow-up of a participant is April 30, 2026, or the date of premature end of follow-up in period 2.

Premature end of follow-up is as follows: death, OR [transfer to another nursing home, return to home or transfer to another care facility] AND no return to the nursing home before the end of the period.

#### Primary analysis

The main analysis will compare the incidence of first severe (leading to oxygen therapy or followed within 30 days by hospitalization or death) ARI between periods when UVGI devices are unfiltered (active) and filtered (inactive). The numerator will be the number of individuals experiencing ≥ 1 incident severe ARI during a given period. Incident ARIs will be those starting > 7 days after baseline. The denominator will be cumulative follow-up time until first severe ARI (if any) or exit (if no ARI occurred) from the period. The result will be expressed as an incidence rate ratio (IRR) with 95% confidence interval (CI).

The analysis will be performed using a generalized linear mixed model (GLMMs) with a Poisson distribution; in cases of overdispersion, a negative binomial model will be used as a robust alternative.

Models will include a log link function; an offset term for person-time; fixed effects for intervention and period; and random effects at the cluster, cluster-period, cluster-individual, and individual levels. The correlation structure will be of the exchangeable block type.

If the individual-level model fails to converge, analyses will be repeated at the cluster level, following the methods described by Hayes & Moulton [28].

#### Sensitivity analyses

Sensitivity analyses will be carried on:

- Using cumulative incidence of severe ARI as the endpoint (total number of ARIs/total follow-up time), with a random effect for individual-periods.
- Adjusting the primary model for baseline covariates: age, sex, and GIR dependency index, measured at the start of each period.
- Defining incident severe IRA as severe ARI episodes starting the following: (i) > 4 days after baseline; (ii) the day after baseline or later.

#### Secondary analyses

##### Clinical secondary outcomes

These secondary analyses will compare the incidence of ARIs of any grade, AEIs, and all-cause hospitalization or death between periods when UVGI devices are unfiltered (active) versus filtered (inactive). The result will be expressed as an incidence rate ratio (IRR) with 95% confidence interval (CI). The statistical framework will mirror that used for the primary endpoint, with adaptations as required by event frequency or distribution.

##### Microbiological secondary endpoints

These secondary analyses will compare microbiological loads in air and fomite samples between periods when UVGI devices are unfiltered (active) versus filtered (inactive). The outcome will be expressed as cycle threshold (Ct) values and number of genome copies/m^3^ for viral agents; and colony-forming units (CFU)/cm^2^ for bacterial and fungal agents. Mean microbial loads will be compared using mixed-effects linear regression models, including random effects for cluster and period.

### Interim analyses {21b}

No interim analyses are planned. The DSMB may request an exploratory parallel comparison (Group A vs. Group B) after completion of the first period to assess early indications of efficacy or futility. If such analysis is conducted, the Haybittle–Peto stopping rule will be applied (*p* = 0.0027). Due to the conservative nature of this threshold, the final analysis significance level will remain *α* = 0.05.

### Methods for additional analyses (e.g. subgroup analyses) {20b}

Exploratory subgroup analyses may be conducted when clinically relevant. Candidate subgroups include age categories, sex, and baseline dependency level (*GIR index*).

Interaction terms between subgroup variables and intervention allocation will be tested first. Only if significant interactions are observed will stratified results be presented. These analyses will be hypothesis-generating and interpreted with caution due to limited statistical power.

### Methods in analysis to handle protocol non-adherence and any statistical methods to handle missing data {20c}

#### Protocol deviations

All randomized centers and residents will be included in ITT analyses unless they opted out. Per-protocol analyses may be performed as sensitivity analyses if warranted.

#### Missing data

For explanatory covariates with < 10% missingness and a verified missing-at-random pattern, multiple imputation by chained equations will be applied. For covariates with > 10% missingness, no imputation will be performed; such variables will be excluded from multivariable models. No imputation will be performed for missing outcome data.

### Plans to give access to the full protocol, participant-level data and statistical code {31c}

The full study protocol and the statistical analysis plan will be made publicly available on ClinicalTrials.gov (identifier: NCT06569160). Participant-level data and statistical code will be made available to qualified researchers upon reasonable request to the corresponding author, after publication of the final study results.

## Oversight and monitoring

### Composition of the coordinating center and trial steering committee {5d}

The Scientific Advisory Board (SAB) comprises the Coordinating Investigator, the trial biostatistician, the Director of the Clinical Trial Unit, the project manager, and three external experts appointed by the sponsor. The latter are selected for their expertise in geriatrics, infectious diseases, public health, or clinical research methodology. The board is chaired by one of the external members, elected by their peers.

The SAB meets twice yearly until study completion. Extraordinary meetings may be convened at the discretion of the Chair, or upon request of the Coordinating Investigator or the sponsor, in response to urgent scientific, ethical, or operational issues.

The SAB oversees the overall conduct of the trial from scientific, ethical, and logistical perspectives. Its responsibilities include:

- Monitoring trial progress and ensuring adherence to the approved protocol.
- Ensuring the continued scientific relevance of the research questions and methodological integrity of the study.
- Overseeing procedures for data access and dissemination of findings.
- Maintaining communication with the sponsor and the Data and Safety Monitoring Board (DSMB) to coordinate oversight activities.
- Reviewing and approving protocol amendments and key operational decisions, including site openings and closures.

### Composition of the data monitoring committee, its role and reporting structure {21a}

The Data and Safety Monitoring Board (DSMB) consists of three independent experts in clinical research, infectious diseases, geriatrics, and biostatistics, none of whom have competing interests or involvement in the trial.

The DSMB reviews interim safety and efficacy data and provides recommendations to the sponsor regarding continuation, modification, or termination of the study. Particular vigilance will be exercised regarding any emerging safety signal, although current evidence indicates that the risk of UVGI-related toxicity is minimal.

The DSMB will also review the appropriateness of the primary endpoint. At the design stage, investigators debated between a broad definition of acute respiratory infections (all grades of severity) and a narrower definition limited to severe lower respiratory infections, owing to the scarcity of recent incidence data for either category. During the trial, the DSMB will examine observed event rates and may, if warranted, recommend modifying the primary endpoint, provided that such changes do not compromise the principle of quadruple blinding.

### Adverse event reporting and harms {22}

No toxicity is expected from the intervention, given the well-established safety profile of UVGI systems. Nevertheless, systematic surveillance will be implemented to detect and document any potential adverse event.

Particular attention will be paid to skin erythema in areas exposed to light and to keratitis, which represent the main theoretical risks associated with UVGI exposure.

All adverse events (AEs) will be documented by the trial’s clinical research assistants and reviewed by the Clinical Event Review Committee (CERC). Events will be classified according to severity using the Common Terminology Criteria for Adverse Events (CTCAE).

Any serious adverse event (SAE), whether expected or unexpected, will be immediately reported by investigators to the sponsor. The sponsor will verify the report and forward validated cases to the relevant French and European pharmacovigilance authorities, in compliance with regulatory requirements.

The DSMB will receive periodic summaries of all AEs and SAEs and will continuously assess safety data throughout the study.

### Frequency and plans for auditing trial conduct {23}

Trial documentation is maintained in a Trial Master File (TMF) in accordance with ICH-GCP standards.

The TMF, study database, and source data may be audited at any time by the sponsor or by independent entities designated by regulatory authorities, to verify compliance with Good Clinical Practice, applicable regulations, and the approved protocol.

### Plans for communicating important protocol amendments to relevant parties (e.g., trial participants, ethics committees) {25}

All protocol amendments approved by the SAB will be submitted to the Ethics Committee that authorized the original protocol. No changes to study conduct will be implemented until formal ethics approval has been obtained.

If the amendment affects the participant information sheet, a revised version will be submitted concurrently for approval. Once validated, the updated information sheet will be provided to all current and future participants.

### Dissemination plans {31a}

At the end of the trial, the main results will be communicated to residents and staff of participating nursing homes through written summaries and oral presentations, using clear, non-technical language adapted for lay audiences.

Findings will be submitted for publication in a peer-reviewed, high-impact journal, and presented at national and international conferences in geriatrics, pneumology, public health, and infection control.

The study is registered on ClinicalTrials.gov, and the final results will be uploaded to the registry.

No publication restrictions apply, aside from adherence to standard authorship guidelines and prior review by the SAB to ensure scientific accuracy and regulatory compliance.

## Discussion

The RESPROTECT trial evaluates the clinical efficacy of UVGI, a long-established environmental intervention which has robust in vitro evidence for microbial inactivation [14, 15], but until recently lacked randomized clinical evidence.

In August 2025, the PETRA team reported the results of a cluster-randomized crossover trial assessing the impact of UVGI on respiratory infections in Australian nursing homes [29]. PETRA yielded mixed findings: no statistically significant difference for its prespecified primary endpoint (incidence of ARIs per 6-week period), but a significant reduction in a post-hoc analysis of cumulative incidence.

RESPROTECT is designed to provide complementary evidence in this area.

Compared with PETRA, RESPROTECT incorporates several features aimed at strengthening internal validity and precision: a larger number of clusters and participants, a higher density of UVGI devices in communal areas, and quadruple blinding.

The selection of severe ARIs as the primary endpoint—defined by objective criteria such as oxygen therapy, hospitalization, or death—maximizes clinical relevance and ensures complete, verifiable ascertainment through electronic health records. In addition to ARIs, RESPROTECT also evaluates all-cause hospitalizations and mortality.

Conducting a multicenter randomized trial in nursing homes presents distinctive logistical and ethical challenges. Operationally, the installation and calibration of UVGI devices demand careful and detailed technical assessments of room geometry. Nursing homes are less accustomed to clinical research than hospitals, requiring extensive preparatory work: early dialogue with leadership, staff engagement, and transparent communication with residents and families to ensure understanding and trust.

Finally, the nursing home environment—characterized by prolonged indoor cohabitation of highly susceptible individuals—constitutes a logical first-line setting to evaluate upper-room UVGI [16, 23, 30]. If RESPROTECT demonstrates clinical benefit, future research should focus on long-term cost-effectiveness, implementation strategies, and potential extension to other high-risk institutional or community settings.

## Trial status

The current protocol version is 3.0, dated September 30, 2025.

Recruitment began on October 1st, 2024. Recruitment and follow-up were completed on April 30, 2026.

## Data Availability

Investigators will have full access to the final dataset. No contractual agreements restrict this access.

## Abbreviations

ARI: Acute respiratory infection
CRF: Case Report Form
CTCAE: Common Terminology Criteria for Adverse Events
DSMB: Data and Safety Monitoring Board
SAB: Scientific Advisory Board
UVGI: Ultraviolet germicidal irradiation
